# Acceptance of immersive head-mounted virtual reality in older adults

**DOI:** 10.1038/s41598-019-41200-6

**Published:** 2019-03-14

**Authors:** Hanne Huygelier, Brenda Schraepen, Raymond van Ee, Vero Vanden Abeele, Céline R. Gillebert

**Affiliations:** 1 Department for Brain and Cognition, KU Leuven, Leuven, Belgium; 2 Donders Institute for Brain, Cognition and Behavior, Radboud University, Nijmegen, The Netherlands; 3 Philips Research, High tech Campus, Eindhoven, The Netherlands; 4 E-media lab, KU Leuven, Leuven, Belgium; 5 Department of Experimental Psychology, University of Oxford, Oxford, United Kingdom

## Abstract

Immersive virtual reality has become increasingly popular to improve the assessment and treatment of health problems. This rising popularity is likely to be facilitated by the availability of affordable headsets that deliver high quality immersive experiences. As many health problems are more prevalent in older adults, who are less technology experienced, it is important to know whether they are willing to use immersive virtual reality. In this study, we assessed the initial attitude towards head-mounted immersive virtual reality in 76 older adults who had never used virtual reality before. Furthermore, we assessed changes in attitude as well as self-reported cybersickness after a first exposure to immersive virtual reality relative to exposure to time-lapse videos. Attitudes towards immersive virtual reality changed from neutral to positive after a first exposure to immersive virtual reality, but not after exposure to time-lapse videos. Moreover, self-reported cybersickness was minimal and had no association with exposure to immersive virtual reality. These results imply that the contribution of VR applications to health in older adults will neither be hindered by negative attitudes nor by cybersickness.

## Introduction

**Figure 1:**
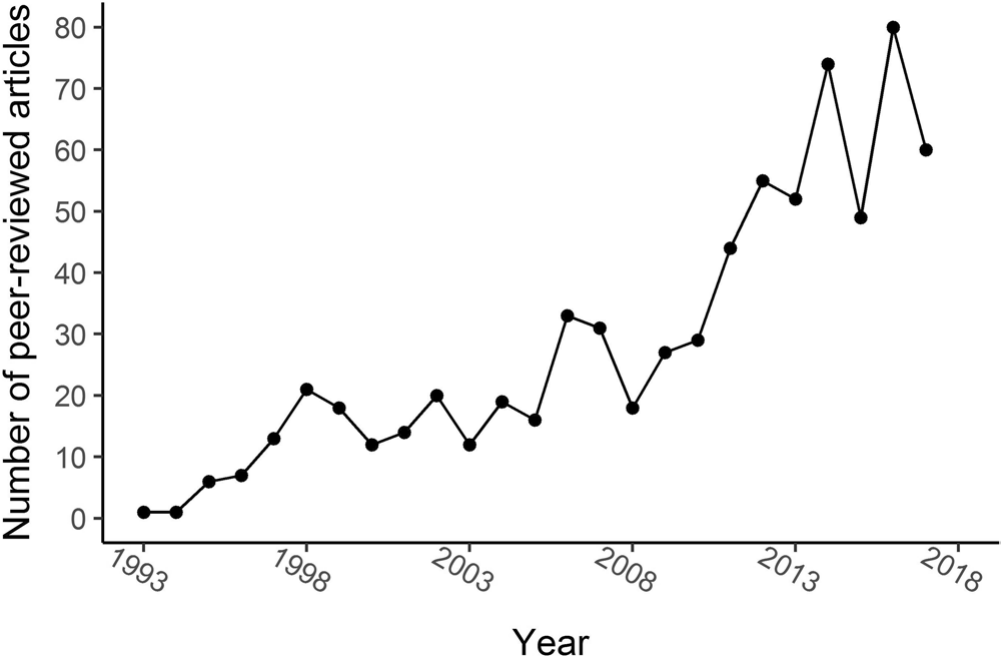
VR health publication trends. Number of peer-reviewed articles in the Scopus database per year based on the query “virtual reality AND health OR assessment OR treatment OR rehabilitation OR recovery”.

*Virtual reality* (VR) has received great interest from the health community, as it offers many opportunities to improve the assessment and treatment of health problems^1–6^. A growing trend of VR health publications is indeed evident (Fig. 1). Within this body of literature, VR is defined in various ways, referring to a vast number of devices and different levels of immersion. Based on Milgram and colleagues’ mixed reality continuum^7^, we define *immersive VR* as fully computer-generated environments where head-mounted displays (HMD-VR, e.g. Oculus Rift) or projection-based systems (e.g. a Cave Automated Virtual Environment^8^) provide a full field of view. Fully computer-generated environments presented on displays with a limited field of view (e.g. a monitor) are labelled *semi-immersive VR*, and *augmented virtuality (AV)* is used to identify systems in which real world information is superimposed onto computer-generated environments^9^ (e.g. Xbox Kinect).

VR health applications frequently target health conditions that are prevalent particularly among older individuals^10^. For instance, AV has been used for post-stroke motor rehabilitation^11–16^ and HMD-VR has been used for memory training in nursing home residents^17^ and for post-stroke cognitive assessment^18,19^. Compared to traditional computerized methods, immersive VR offers the opportunity to assess and train cognition in a more sensitive, ecologically valid and safe way^1,2,5^. Moreover, head-mounted devices offer optimal control over sensory stimuli resulting in easy standardization of the testing conditions (e.g. in terms of viewing distance, luminance), which is likely to enhance the reliability of cognitive assessment^20^. Until recently, the development of VR health applications often relied on custom-made devices that were not broadly available to others. The new head-mounted immersive VR devices (e.g. Oculus Rift, HTC Vive) are expected to boost the widespread use and development of VR^1^. The contribution of head-mounted immersive VR to health care may however be hindered by the end-users’ attitudes towards HMD-VR as well as by cybersickness^21,22^.

Since technology usage depends on technology attitudes^23^ and older adults have more negative attitudes towards new technology^24,25^, it is important to understand attitudes towards HMD-VR in this target group. According to the *unified theory of acceptance and usage of technology (UTAUT)*^23^, the intention to use technology or *technology acceptance* is influenced by the perceived usefulness of technology (*performance expectancy*) and the perceived ease of using technology (*effort expectancy*). When combined, performance and effort expectancy are also referred to as *attitude*^23^. A recent meta-analysis revealed that technology acceptance was negatively associated with chronological age and that this association was fully mediated by performance and effort expectancy^25^. Although studies based on UTAUT or related models clarify how attitudes affect technology usage in older adults^26^, they give no insight into age- or generation-associated antecedents of technology attitudes^27^. Previous studies have revealed the importance of experience with technology on technology attitudes and adoption^28–32^. Moreover, although technology usage has a negative association with intelligence^33^, the potential influence of global cognitive decline on technology attitudes in older adults has received less attention than the influence of technology experience. Mild cognitive impairment is associated with difficulties in managing one’s daily life^34,35^, which in turn may result in a reduced willingness to undertake the challenge of learning to use new technology. Global cognitive impairment may therefore be associated with more negative technology attitudes. Importantly, researchers who develop, and clinicians who wish to use immersive VR health applications for older adults, will need to take into account the acceptance of immersive VR in this population.

Although the efficacy of VR health applications has been studied in diverse clinical populations^11–16,36^, the assessment of the user acceptance, experience and safety of these approaches is often limited^37^. It has been evaluated whether stroke survivors are more sensitive to cybersickness due to HMD-VR exposure than age-matched healthy controls in a small sample by Kang and colleagues^21^ and whether objective performance in an HMD-VR driver simulation was associated with subjective comfort level by Simone and colleagues^38^. These studies revealed that stroke patients were equally sensitive to cybersickness than neurologically healthy participants and that objective performance in a HMD-VR driver simulation was not associated to subjective comfort level. A different study showed that a projection-based immersive VR system was generally accepted by older adults with cognitive impairments, as 68% of participants with mild cognitive impairment or dementia preferred an immersive VR visual search task above a similar pen- and paper task^39^. Participants preferring the VR task reported it to be more engaging and immersive than the pen- and paper task. However, some participants preferred the pen- and paper task, and reported that it was easier to use, looked more familiar to them and was less tiring for their eyes as compared to the immersive VR task^39^. This study suggests inter-individual differences in the acceptance of immersive VR in older adults, but does not provide insight into the characteristics of older individuals predicting these differences. Furthermore, to our knowledge, no studies have reported on the acceptance of HMD-VR in older populations.

**Figure 2:**
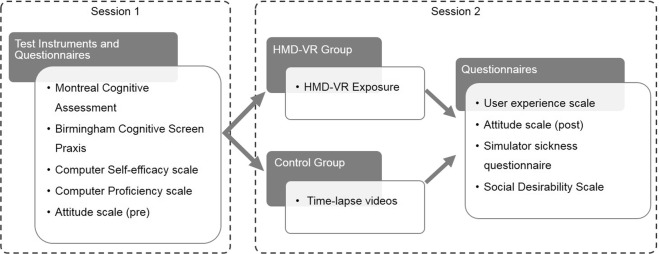
Schematic representation of the study protocol including the order of the test instruments and questionnaires that were administered to participants. In the first session, all participants completed the Montreal Cognitive Assessment^40^, the praxis scale of the Birmingham Cognitive Screen^43^, computer self-efficacy, computer proficiency and attitude towards HMD-VR scales. In a first recruitment phase, participants were allocated to the HMD-VR group (n = 38). In a second recruitment phase, participants (n = 38) were allocated to the control group. The two groups were matched on age, education, gender and independent living status. After exposure to HMD-VR or time-lapse videos in a second session, the user experience of the HMD-VR or time-lapse video condition was measured. Afterwards participants completed a second administration of the attitude scale and completed the simulator sickness questionnaire^41^. A subset of 44 participants also completed the Marlowe-Crowne social desirability scale^42^.

Given the popularity of VR for health applications for older adults and the lack of knowledge on acceptance of HMD-VR in older adults, we investigated the attitudes of older adults towards HMD-VR. We evaluated whether attitudes changed after a first HMD-VR exposure and whether this attitude change was associated with how participants experienced the HMD-VR exposure. For this purpose, we compared post-pre attitude differences between a group of participants exposed to HMD-VR and a control group exposed to time-lapse videos presented on a standard notebook computer (Fig. 2). Attitudes were measured with a scale containing questions gauging the perceived ease of use, the perceived usefulness and the  willingness to use HMD-VR. We also measured how participants experienced these activities using a *user experience* scale that contained items gauging the users’ enjoyment and immersion.

Additionally, we hypothesized that age would have a negative association with initial attitudes towards HMD-VR (attitudes prior to the HMD-VR or video exposure), similarly to what has been shown before^24–26^. Given the hypothesis that the negative age-attitude association relates to a generation-related lack of technology experience^28–30^, we predicted that *computer proficiency* would mediate the relation of age and initial attitudes. Computer proficiency was measured by letting participants rate their ability to perform beginner and advanced computer activities. In addition, previous studies identified *computer self-efficacy* as an important mediator of age and technology usage^33^, and therefore we predicted that computer self-efficacy would also mediate the relation of age and initial attitudes. Computer self-efficacy was measured by letting participants rate their confidence and anxiety in performing computer activities. We additionally included *global cognitive status* as a third potential mediator of the association between age and initial attitudes, given that previous literature showed that cognitive abilities were related to technology adoption^33^. Global cognitive status was measured with the Montreal Cognitive Assessment (MoCA) on which scores range from 0 to 30, and scores below 26 indicate mild cognitive impairment^40^.

Attitudes, user experience and computer self-efficacy were measured with scales consisting of 5-point Likert rated items in which 3 represented a neutral position, 1 represented the lowest score, and 5 represented the highest score. We also compared self-reported *cybersickness* between the HMD-VR and control group using the Simulator Sickness Questionnaire (SSQ)^41^, and checked the effect of social desirability on the initial attitudes using a short version of the Marlowe-Crowne Social Desirability Scale^42^. Finally, we measured the ability of the participants to execute purposeful actions with their upper limbs using the praxis subscale of the Birmingham Cognitive Screen^43^, and registered their level of independence in activities of daily living and their experience with technology (e.g. the number of digital devices they have used). Analyses were performed with frequentist statistics, complemented by *Bayes Factors* (BF) to quantify the relative strength of evidence in favor of the null or alternative model. A BF_01_ represents the strength of evidence in favor of the null hypothesis, while a BF_10_ represents the strength of evidence in favor of the alternative hypothesis^44^.

## Results

### Participants

Seventy-six volunteers between ages 57 and 94 years participated in this study. Half of the participants (n = 38) was allocated to the HMD-VR group and the other half was allocated to the control group. One participant of the HMD-VR group dropped out after the first session for an unknown reason. One participant of the control group refused further participation during the first session (prior to completing a single test or questionnaire) and was replaced by a new participant to complete the sample of 38 individuals. The HMD-VR and control group were matched on age (BF_01_ = 4.2) according to a Bayesian independent samples t-test, and matched on education level (BF_01_ = 8.6) according to a Bayesian contingency table test. The groups were also matched on gender and independent living status. Participant characteristics of both groups are reported in Table 1. More details on participant recruitment are reported in the Methods section.

**Table 1:** Participant characteristics of the HMD-VR and control group.

Descriptive variable	HMD-VR (n = 38)	Control (n = 38)
Age M (SD, Min – Max)	74.8 (10.4, 60–92)	74.8 (12.2, 57–94)
Sex (Male/Female)	19/19	19/19
Education (Low/Mid/High)	12/12/14	12/12/14
Handedness (Left/Right)	6/32	4/34
Computer use in hours M (SD)	2.0 (2.1)	1.1 (1.8)
Number of digital devices used M (SD)	0.9 (0.3)	1 (0)
Number of tasks performed with a digital device M (SD)	0.9 (0.3)	1 (0)
Frequency of playing digital games (never/at least once)	17/21	27/11
Used an Xbox game controller (never/at least once)	35/3	36/2
Heard of virtual reality before study (never/at least once)	18/20	11/27
MoCA total score M (SD, Min – Max), failed/passed	24.0 (4.8, 10–30), 21/17	22.8 (5.6, 9–30), 22/16
BCoS Praxis complex figure copy (failed/passed)	4/34	8/29^a^
BCoS Praxis multi-step object use (failed/passed)	5/33	4/34
BCoS Praxis gesture production (failed/passed)	1/37	1/37
BCoS Praxis gesture recognition (failed/passed)	0/38	3/35
BCoS Praxis imitation (failed/passed)	2/36	1/37
Receives help for food preparation (no/yes)	19/19	22/16
Receives help for medication use (no/yes)	21/17	28/10
Receives support in housekeeping (no/yes)	15/23	18/20

M = mean, SD = standard deviation, Min = minimum, Max = maximum, Education: Low corresponds to years of formal education <=6, mid corresponds to years of formal education between 7–12 and high corresponds to years of formal education >12. BCoS Praxis = praxis subscale of the Birmingham Cognitive Screen^43^, MoCA = Montreal Cognitive Assessment^40^, failed = performance was below the 5^th^ percentile cutoff score reported in the test manual of the BCoS Praxis or below the cut-off score of 26 on the MoCA, passed = performance was above the 5^th^ percentile cutoff score reported in the test manual of the BCoS or above or equal to a score of 26 on the MoCA. ^a^One participant of the control group did not complete this task.

### Changes in attitudes after a first HMD-VR exposure

**Figure 3:**
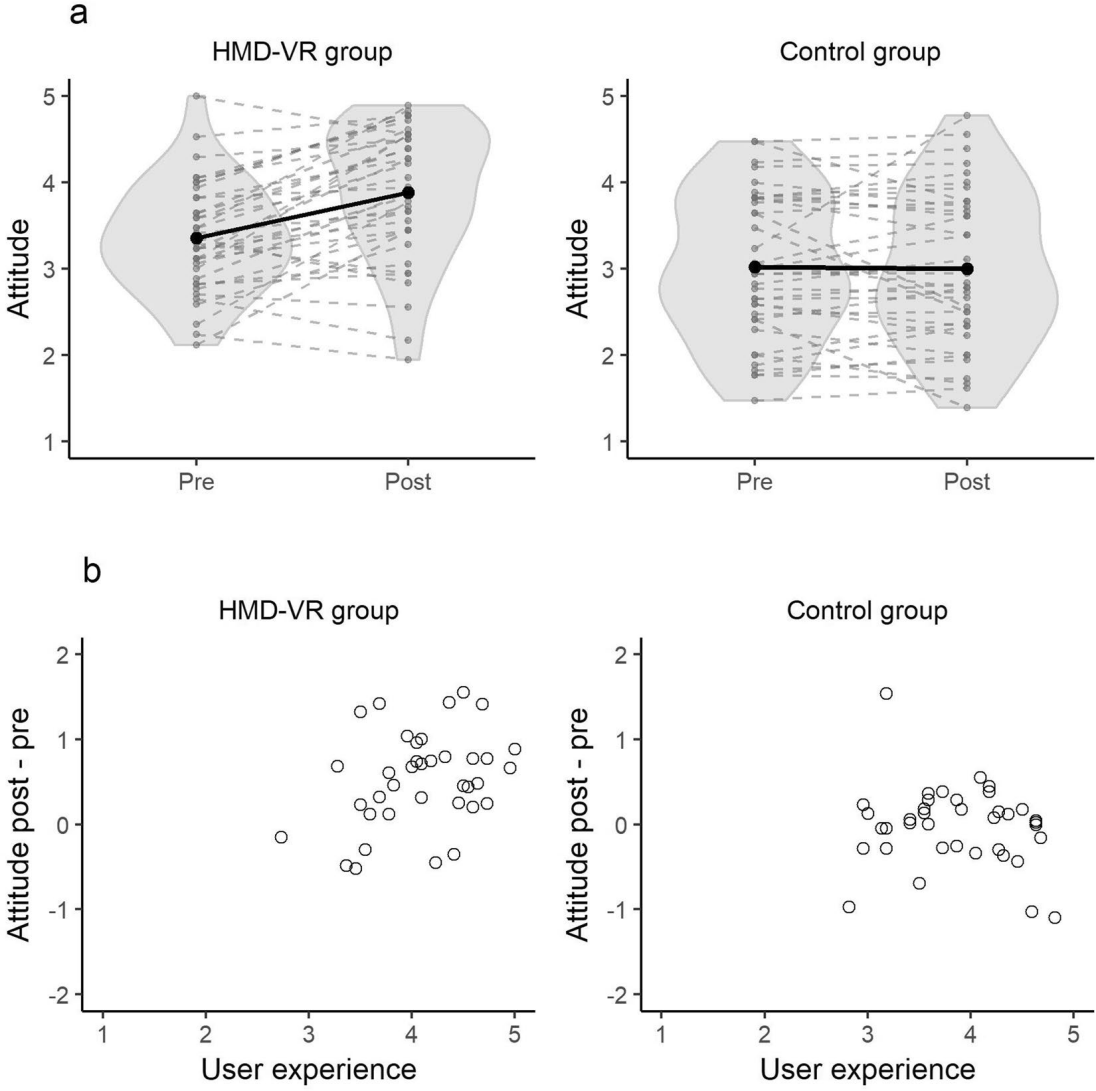
Attitudes towards HMD-VR. (**a**) depicts the mean score on the attitude scale of each participant on the pre- and post-assessment in the HMD-VR and control group. Each dashed grey line represents the observed scores of one participant, while the black solid line represents the group average. The grey area represents the density plots of the observed mean attitude scores. The results show a positive trend in the HMD-VR group and a stable trend in the control group. (**b**) depicts the relation between the attitude difference between the post- and pre-assessment as a function of the mean score on the user experience scale for the HMD-VR and control group. Each dot represents the observed mean score of one participant. The results suggest a positive relation between self-reported user experience and attitude difference in the HMD-VR group but not in the control group.

We observed neutral attitudes towards HMD-VR prior to a first exposure to HMD-VR. In the HMD-VR group, attitudes increased from 3.4 (SD = 0.6) to 3.9 (SD = 0.8) after a first HMD-VR exposure, while in the control group initial attitudes were neutral (M = 3.0, SD = 0.8) and remained neutral after time-lapse video exposure (M = 3.0, SD = 0.9) (Fig. 3a). According to an analysis of covariance (ANCOVA) that modeled the post-pre attitude difference as a function of group (HMD-VR vs. control) and the self-reported user experience, there was inconclusive support for a main effect of group on the attitude difference (F(1, 71) = 2.56, *P* = 0.11, BF_10_ = 1.02). There was also inconclusive evidence for a main effect of self-reported user experience (F(1, 71) = 4.49, *P* = 0.04, BF_10_ = 1.01). Finally, there was anecdotal evidence for an interaction between self-reported user experience and group (F(1, 71) = 5.0, *P* = 0.03, BF_10_ = 2.6). The interaction between self-reported user experience and group suggests that a more positive self-reported user experience was associated with a larger post-pre attitude difference in the HMD-VR group, but not in the control group (Fig. 3b). Levene’s test indicated no significant heteroscedasticity, (F(1, 73) = 2.15, P = 0.15) and visual inspection of residuals showed no violations of the ANCOVA assumptions (Supplementary Materials 1).

### Factors predicting initial attitudes

**Figure 4:**
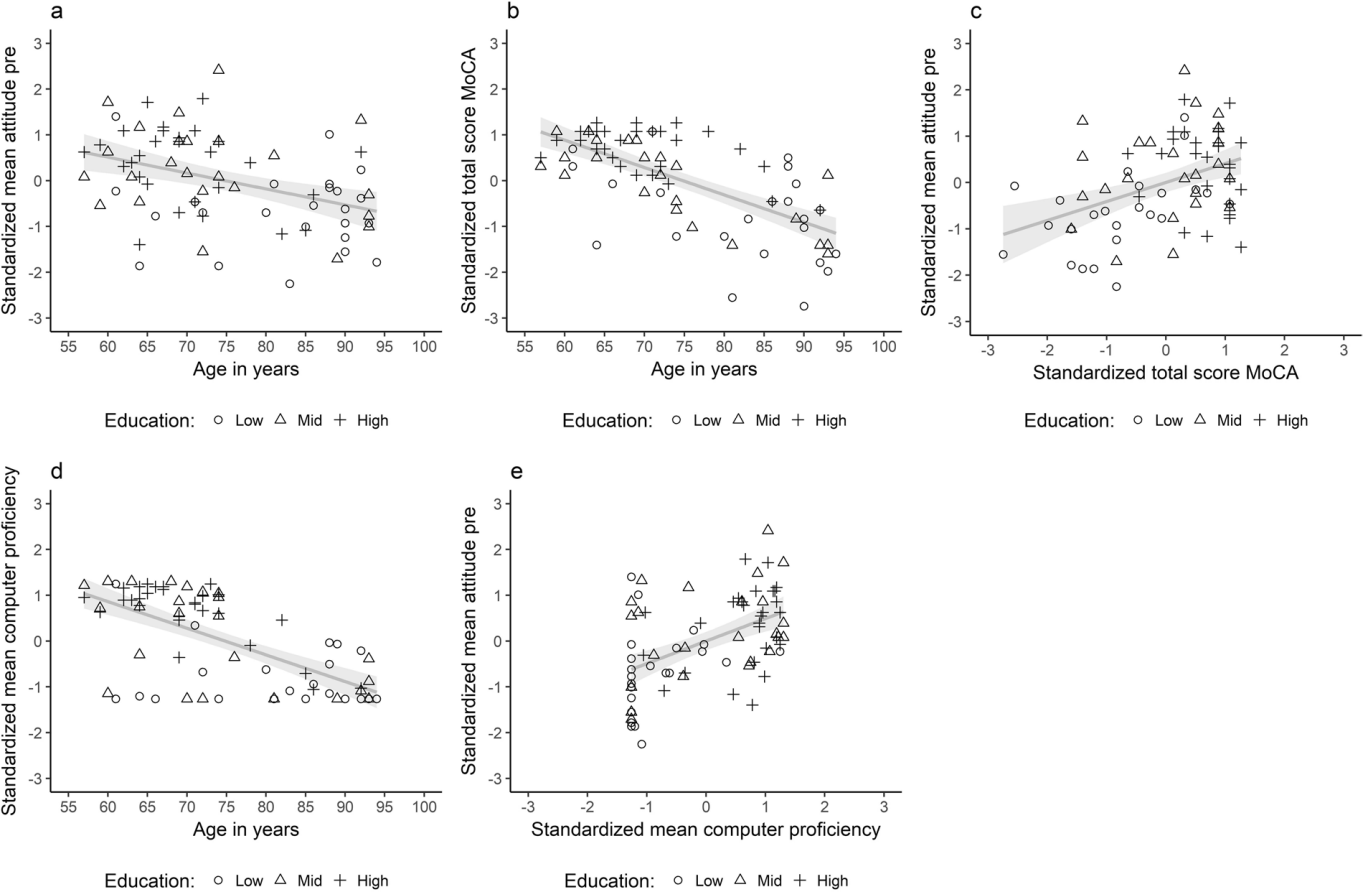
Pairwise scatterplots for all variables included in the path analysis. Shapes represent the three different levels of education, while years of formal education were included in the path model. A low education level corresponds to years of formal education ≤6, mid education level corresponds to years of formal education >7 and ≤12 and high education level corresponds to years of formal education higher than 12.

**Figure 5:**
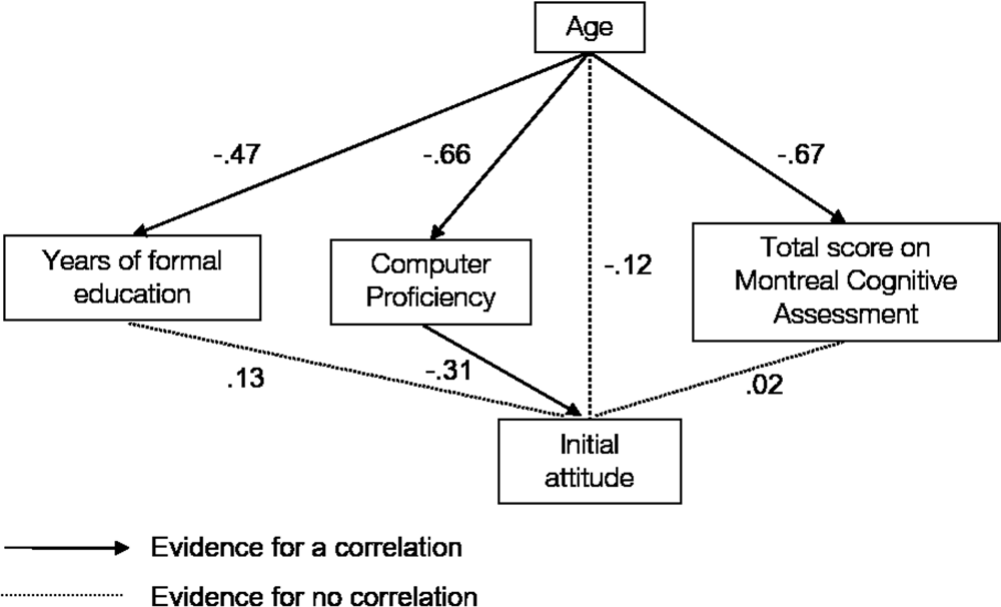
Predictors of initial attitudes. The standardized regression coefficients for each path are depicted. The association of age with computer proficiency, education and the MoCA were significant. There was no residual association of age and attitudes corrected for computer proficiency, education and MoCA. There was no residual association of years of formal education and attitudes corrected for computer proficiency, age and MoCA. There was no residual association of MoCA and attitudes corrected for years of formal education, computer proficiency and age. The association between age and attitudes was not mediated through education and MoCA. The mediation role of computer proficiency for the relation between age and attitudes was inconclusive.

A path model was estimated to study the association between age and initial attitudes and the extent to which this association was mediated by global cognitive status, computer proficiency and years of formal education. The pairwise correlations and descriptive statistics of all variables considered for the path model of all 76 participants are reported in Table 2. The variables included in the model and results of the model are visualized in Figs 4 and 5. The model could explain 23% of variance in initial attitudes, 43% of variance in computer proficiency, 45% of variance in the MoCA and 22% of variance in years of formal education. Computer proficiency (β = −0.66, *P* < 0.001, BF_10_ = 700,139.5, Fig. 4d), MoCA (β = −0.67, *P* < 0.001, BF_10_ = 33,695,113.2, Fig. 4b) and years of formal education (β = −0.47, *P* < 0.001, BF_10_ = 866.53) were each significantly associated with age. There was strong support for the absence of a residual correlation between initial attitudes and the MoCA according to the BF when correcting for years of formal education, computer proficiency and age (β = 0.02, *P* = 0.90, BF_01_ = 11.0, 95% CI [−0.25, 0.28], Fig. 5). In addition, the BF supports the hypothesis of no residual correlation between initial attitudes and years of formal education when correcting for age, computer proficiency and the MoCA score (β = 0.13, *P* = 0.26, BF_01_ = 6.15, 95% CI [−0.09, 0.35], Fig. 5). There was evidence for a residual correlation between initial attitudes and computer proficiency (β = 0.31, *P* < 0.001, BF_10_ = 3.96, 95% CI [0.06, 0.57]), and there was no residual association between initial attitudes and age when correcting for computer proficiency, MoCA and years of formal education (β = −0.12, *P* = 0.49, BF_01_ = 6.8, 95% CI [−0.44, 0.21]). There was substantial support for the absence of mediation of age and initial attitudes by the MoCA (β = −0.01, *P* = 0.90, BF_01_ = 11.01, 95% CI [−0.19, 0.17]) and by years of formal education (β = −0.06, *P* = 0.27, BF_01_ = 9.72, 95% CI [−0.17, 0.05]) according to the BF. The mediation of age and initial attitudes by computer proficiency was unclear as it was significant at the 0.05 level (β = −0.21, *P* = 0.02, 95% CI [−0.38, −0.03]), but the BF favors the null hypothesis (BF_01_ = 2.3).

**Table 2:** Pairwise correlations, descriptive statistics and 95% bootstrapped confidence intervals of all variables considered for the path analysis.

Variable	Age	CP	CSE	MoCA	YFE	In attitude
Age		[−0.77, −0.49]	[−0.68, −0.33]	[−0.77, −0.56]	[−0.62, −0.32]	[−0.58, −0.22]
CP	−0.65***		[0.78, 0.90]	[0.65, 0.80]	[0.63, 0.80]	[0.25, 0.65]
CSE	−0.52***	0.85***		[0.44, 0.68]	[0.44, 0.67]	[0.28, 0.62]
MoCA	−0.67***	0.74***	0.58***		[0.54, 0.73]	[0.19, 0.55]
YFE	−0.47***	0.72***	0.55***	0.66***		[0.24, 0.56]
In attitudes	−0.39***	0.49***	0.48***	0.41***	0.42***	
M	74.8	3.0	2.9	23.4	12.3	3.2
SD	11.3	1.6	1.0	5.0	3.3	0.8

***Significant at the 0.001 level after Holms correction for multiple comparisons. CP = computer proficiency, CSE = computer self-efficacy, MoCA = total score on the Montreal Cognitive Assessment, YFE = years of formal education, In attitudes = mean score on the first administration of the attitude scale, M = mean, SD = standard deviation. Computer self-efficacy was not included in the model, since it was strongly correlated with computer proficiency and would result in multicollinearity (>0.80). We did include years of formal education, as it was only moderately correlated with age, and we wanted to dissociate the effects of age and education.

### Cybersickness

None of the participants in the HMD-VR group reported any severe discomfort on the SSQ scale, while two participants reported one severe discomfort and one participant reported two severe discomforts in the control group. The proportions of participants reporting moderate, mild or no complaints for each SSQ item in the HMD-VR and control group were low and similar between both groups (Table 3). There were more mild and moderate complaints in the HMD-VR group on some of the single symptoms than in the control group (Table 3), but exploratory analyses showed no significant differences at the single-item level of the SSQ (all uncorrected p-values larger than 0.13, Supplementary Materials 2). Across all SSQ items, there was substantial evidence for independence between self-reported discomfort and the participant group for severe (BF_01_ = 24.7, 95% CI probability difference [−0.05, 0.20]), moderate (BF_01_ = 238, 95% CI probability difference [−0.13, 0.25]), mild (BF_01_ = 76.06, 95% CI probability difference [−0.32, 0.08]) and no complaints (BF_01_ = 42.02, 95% CI probability difference [−0.10, 0.05]).

**Table 3:** Proportion of participants that reported moderate, mild or no discomfort on the SSQ.

SSQ item	Moderate		Mild		No	
	HMD-VR	Control	HMD-VR	Control	HMD-VR	Control
General discomfort	0.00	0.05	0.11	0.16	0.89	0.79
Fatigue	0.05	0.11	0.08	0.13	0.87	0.76
Headache	0.00	0.00	0.05	0.09	0.95	0.92
Eye strain	0.05	0.08	0.16	0.09	0.78	0.82
Difficulty focusing	0.00	0.08	0.19	0.09	0.81	0.82
Increase of salivation	0.00	0.03	0.05	0.00	0.95	0.97
Sweating	0.03	0.03	0.16	0.03	0.81	0.92
Nausea	0.00	0.00	0.03	0.03	0.97	0.97
Difficulty concentrating	0.03	0.08	0.08	0.16	0.89	0.74
Fullness of the head	0.03	0.13	0.14	0.08	0.84	0.76
Blurred vision	0.03	0.08	0.08	0.08	0.89	0.84
Dizziness with eyes open	0.00	0.00	0.03	0.03	0.97	0.97
Dizziness with eyes closed	0.00	0.05	0.00	0.03	1.00	0.92
Vertigo	0.00	0.03	0.00	0.05	1.00	0.92
Stomach awareness	0.00	0.00	0.00	0.05	1.00	0.95
Burping	0.00	0.00	0.03	0.03	0.97	0.97

Values represent the proportion of participants who rated each SSQ item as moderate, mild or no discomfort. Ratings of severe discomfort on the SSQ items are not included in the table as they did not occur in the HMD-VR group.

## Discussion

Given the rising popularity of VR for the assessment and treatment of a variety of health conditions that are common in older adults^1–6^, and the emergence of new low-cost high-quality immersive head-mounted displays, it is important to understand acceptance of HMD-VR in this population. In this study, we investigated older adults’ attitudes towards HMD-VR and assessed changes in attitudes after a first HMD-VR exposure. We tested older adults of a broad range of ages, education levels, technology experience, global cognitive statuses and levels of independence (Table 1), as we assumed that these participants may be more representative of many immersive VR health applications’ end-users than samples mainly consisting of community dwellers or baby-boomers.

The results showed that older adults without prior experience with HMD-VR had a neutral attitude towards this new technology. This result corresponds to previous findings on technology attitudes in older adults^26,45^. Furthermore, we found no association between initial attitudes towards HMD-VR and social desirable response styles in a subgroup who completed a social desirability scale (Supplementary materials 3). In addition, we found evidence that attitudes became more positive after a first exposure to HMD-VR, while attitudes remained stable in a group of participants who watched time-lapse videos on a standard notebook computer. This interaction effect suggests that the change in attitudes in the HMD-VR group was not merely the result of a positive experience, but was due to HMD-VR exposure itself. This result is compatible with previous findings on the effect of computer experience on computer attitudes^31^. The observation that attitudes towards HMD-VR can be improved by a first HMD-VR exposure further supports the use of HMD-VR in the older population and suggests that HMD-VR acceptance in the current cohort of older adults should not necessarily be of concern to HMD-VR health application developers. However, future studies are needed to reveal which features of the HMD-VR application itself and which methods of introducing older adults to HMD-VR applications affect the acceptance and user experience of HMD-VR. In addition, the type of help needed while using HMD-VR and the extent to which older adults can learn to use HMD-VR independently remains an important question.

In addition, we explored whether certain characteristics of older individuals predicted initial attitudes towards HMD-VR. Previous research revealed that computer interest was associated with age, education, and computer knowledge^32^, that technology usage depended on age and that the latter was mediated by computer anxiety and intelligence^33^. Our results expanded this knowledge base by investigating the relationship between age, attitudes towards HMD-VR and the mediating role of computer proficiency and global cognitive status in a heterogeneous sample of community dwellers and adults in assisted living. As predicted, age had a significant negative association with initial attitudes without correcting for computer proficiency, global cognitive status and years of formal education, which is in line with earlier findings^25^. Moreover, the age-attitude association was not mediated by global cognitive status when controlling for computer proficiency and years of formal education. Thus, although older adults experiencing cognitive decline may experience difficulties in learning to use new technology^33^, our results suggest that they are equally willing to try out new technology as their peers with a better cognitive status.

Cybersickness symptoms did not occur frequently in our sample of participants, and, importantly, were not significantly associated with the exposure to HMD-VR in a between-subject design. Furthermore, although there were more reports of mild to moderate complaints of certain cybersickness symptoms in the HMD-VR than the control group (Table 3), none of these differences were statistically significant (Supplementary Materials 2). Noteworthy is that research in healthy participants and vestibular patients has shown a decline in motion sickness susceptibility with increasing age^46^. More research is needed to clarify how age affects susceptibility to motion sickness induced by different types of HMD-VR applications.

We did not observe major safety concerns when applying HMD-VR in older adults following the HMD-VR device’s safety guidelines. However, we must note that this does not imply that all HMD-VR applications are safe for these end-users. Foremost, we did not systematically assess in what proportion of older adults HMD-VR can be safely used (e.g. proportion of older adults meeting the safety exclusion criteria). The safe applicability of the technology in a large proportion of older adults is an important consideration for HMD-VR health application developers and should be further investigated. Additionally, we cannot make statements about falling risks as participants remained seated during the HMD-VR exposure. It is known that HMD-VR affects dynamic balance in young adults without balance disorders^47^, making it likely that HMD-VR health applications for older adults may best be developed to be used in a seated position. Furthermore, prevalence of cybersickness symptoms depends on the design of the HMD-VR application, with more severe cybersickness symptoms when motion is not under control of the observer^48^. In this study, we used an HMD-VR application with motion that is under control of the observer to minimize the chance of observing certain cybersickness symptoms. Therefore, future research should investigate to what extent older adults are sensitive to motion that is not under their control and how the design of the HMD-VR device and application could mitigate possible cybersickness issues in older adults.

To conclude, we showed that older adults are willing to use HMD-VR and have more positive attitudes towards HMD-VR after a first positive HMD-VR experience. Moreover, the negative association between age and initial attitudes was not mediated through cognitive status or years of formal education. Furthermore, cybersickness was not significantly associated with HMD-VR exposure. These results support the development and use of HMD-VR health applications for older adults.

## Methodology

### Participants

Participants were recruited through nursing homes and participant contact lists of previous studies of our research group. A medical implant or epilepsy were grounds for exclusion based on the HMD-VR device’s EU safety regulations. Poor vision or hearing that could not be corrected by glasses or a hearing aid, inability to provide informed consent and previous HMD-VR exposure were also grounds for exclusion. In the first recruitment phase of the study, all participants were allocated to the HMD-VR group (n = 38). Afterwards, in a second recruitment phase of the study, 38 participants were recruited and allocated to the control group. The participants of the control group were matched on age, education, gender and independent living status to the participants of the HMD-VR group. In each group, 45% of participants were residing in assisted living, while 55% were community dwellers. The majority of participants (89%) had no knowledge that the study would involve technology, and 74% of participants were not recruited by any means of technology (e.g. no e-mail contact, no online advertisements). All study procedures were approved by the Social and Societal Ethics Committee of the KU Leuven (G-2017 01 733) and executed in accordance with the committee’s ethical guidelines. Each participant provided written informed consent.

### Instruments

#### HMD-VR exposure and time-lapse videos

The HMD-VR group experienced the application Perfect of nDreams^49^ using the Oculus Rift and Touch Controllers. By means of the Touch Controllers, the user can interact with the HMD-VR environment, for instance by picking up or throwing objects. The environment is artificially made, but the scenes look natural and familiar (e.g. mountain, lake). This HMD-VR application has no observer-independent background motion in the scenes. The HMD-VR exposure was audio-taped. The control group watched six pre-selected time-lapse nature videos on the YouTube broadcasting system available under the standard YouTube License. While watching the videos they listened to the audio through headphones to mimic wearing a head-mounted device in the HMD-VR condition. The videos were selected to resemble the scenery of the HMD-VR environment, to elicit a similar aesthetic appreciation as the HMD-VR environment, and to have an equal duration as the HMD-VR exposure.

#### Neuropsychological Assessment

The MoCA^40^ is a short, sensitive screen for mild cognitive impairment that consists of pen-and-paper tasks measuring executive functions, memory, language and reasoning. The MoCA has good internal consistency (α = 0.83)^40^. The BCoS Praxis measures the ability of participants to execute purposeful actions with their upper limbs^43^. The percentage of exact score agreement from test to re-test ranges from 50% to 60% in neurologically healthy controls^50^. The subscale consists of multistep object use, figure copying and gesture production, recognition and imitation. All tasks were administered and scored according to the test manual instructions, and scores were compared to the age-adjusted cutoff scores of the test manual^50^.

#### Questionnaires

As to date, there is no validated Dutch questionnaire to measure attitudes towards HMD-VR. Thus, we developed an *attitude scale* consisting of questions gauging the perceived ease of use, perceived usefulness and anticipations about using HMD-VR. Eighteen items were constructed: six based on a questionnaire assessing attitude towards computers^51^, five on a questionnaire assessing attitude towards internet^52^ and seven new items were added (Supplementary Table S2).

To measure *user experience*, a 23-item scale gauging enjoyment and immersion was designed (Supplementary Table S3). Six of the 11 enjoyment items were based on the intrinsic motivation inventory^53^ and 10 of the 11 immersion items were translations of the International Test Commission Sense of Presence Inventory items^54^.

*Computer proficiency (CP)* (Supplementary Table S4) was measured with a 22-item scale of which 10 new items were added onto 12 items that were translations of the CP items of Boot and colleagues^55^.

*Computer self-efficacy (CSE)* (Supplementary Table S5) was measured with a 14-item scale gauging the confidence and anxiety in performing beginner and advanced computer activities. Eleven items were direct translations of the items reported in Barbeitte and Weiss^56^.

In addition, we measured *openness* with the 12-item openness subscale of the validated Dutch Neuroticism Extraversion Openness Five-Factor Inventory 3^57^. This scale was excluded from further analyses given its insufficient internal consistency (Supplementary materials 4).

The attitude, user experience and computer self-efficacy scale items were rated on a 5-point Likert scale ranging from “totally disagree” (1) to “totally agree” (5), with a neutral position (3). All items were scored in such a way that the mean score on each scale ranges from a low score of “1” to a high score of “5”. The computer proficiency items were rated on a 5-point scale going from “I have never tried to do this task” (1) to “very easy” (5).

*Cybersickness* was measured with the Simulator Sickness Questionnaire (SSQ)^41^ that was translated by our research team. Each of the 16 SSQ items were rated with four levels representing no, mild, moderate or severe discomfort.

*Social desirability* was measured with a validated short version of the Marlowe-Crowne Social Desirability Scale (SDS)^42^, which was translated by our research team. Each SDS item is designed to elicit a socially desirable response and has to be evaluated as true/untrue by the participant. The total score is the proportion of items on which the participant responded socially desirable.

### Procedure and design

The study consisted of two sessions with on average one day in between the two sessions (range: 0–5 days, Fig. 2). The first session took approximately 60 minutes and the second session took approximately 90 minutes. In the first session, a demographic interview, the MoCA and BCoS Praxis were administered. Then, participants completed the CSE, CP, attitudes and NEO-FFI 3 openness scales. In the second session, the HMD-VR group first received an explanation about the HMD-VR device and how they could interact with the virtual environment. Then, they were exposed to the virtual environment for an average of 26 minutes (SD = 5.5 minutes, range: 8–36 minutes) and participants were allowed to take breaks. After a few minutes of free exploration in the virtual environment, the experimenter assisted participants to let them perform interactions with the virtual objects (e.g. throwing a stone in the lake). Assistance could take the form of reminding participants what they could do in the environment, explaining participants how they could perform actions with the touch controllers, and manually assisting participants to execute these actions. The examiner explained participants how to perform actions on average 5 times (SD = 3, range: 1–18) and manually assisted participants on average 1 time (SD = 3, range: 0–17) during the HMD-VR exposure. Participants in the control group also received an explanation about the HMD-VR device, but were not exposed to HMD-VR. Instead, they watched time-lapse videos. In both groups, the experimenter stayed with the participants and participants were allowed to interact with the experimenter freely and ask as much help as needed to operate the HMD-VR or video application. The participants remained seated at all times. After exposure to the HMD-VR or time-lapse videos, participants were asked about their experience using the user experience scale. The attitude scale was re-administered after the user experience scale, thereafter followed by the SSQ. The SDS was added to the study protocol for the last 44 participants (6 of the HMD-VR and 38 of the control group). All questionnaires were administered with pen and paper as a semi-structured interview supervised by a trained clinical psychologist and in the same order for each participant. Special care was taken to ensure that all participants, including participants with mild cognitive impairment (MoCA < 26) understood each question. In a supplementary analysis on the response style of the participants to the questionnaires, we did not find evidence that participants with mild cognitive impairment had issues understanding the questionnaire items (Supplementary materials 3).

### Data analysis

The dataset and data analysis scripts are available on FigShare^58,59^. Data analysis was performed in R^60^. First, an item analysis to detect items that negatively impacted the scales’ reliability was conducted. Items with an item-total correlation lower than 0.20 were removed. Based on the remaining items, the Cronbach’s alpha, its 95% confidence interval (CI) and the mean inter-item correlation were calculated^61^. If a scale had a Cronbach’s alpha lower than 0.75, the scale was excluded from further analyses^61^.

To test whether a first HMD-VR exposure affected HMD-VR attitudes, we used an ANCOVA to model the post-pre attitude difference as a function of the main effect of the between-subject factor (HMD-VR versus control group), the main effect of the covariate self-reported user experience and the interaction between both variables using the lm function in R^62^. Type III sum of squares were estimated. Thus, we tested whether there was substantial evidence for an effect of group on top of the self-reported user experience and vice versa with inclusion of the interaction term and we tested whether there was substantial evidence for an effect of the interaction term in addition to the two main effects. The evidence for an effect was evaluated based on the BF, which was computed with the Bayes Factors package^63^ according to the same model comparisons used for computing the F-statistics. A BF_10_ larger than 3 was considered substantial evidence in favor of the alternative hypothesis. The assumption of heteroscedasticity was checked with Levene’s test and we visually inspected the association between the fitted values and residuals of the ANCOVA model (Supplementary Fig. S1).

To assess whether initial attitudes were associated with age and whether this association was mediated through years of formal education, computer proficiency and MoCA, we conducted a path analysis. For predictors that had strong collinearity with each other (>0.80), we chose to include only one predictor. Standardized regression coefficients, accompanying 95% CIs and BFs based on the method described in Wetzel and Wagenmakers^64^ were estimated. Path analysis was performed with the Lavaan package^65^. Power analysis in G*Power 3.10^66^ revealed that 60 participants were needed to detect correlations of 0.40 evaluated at a threshold of 0.01 to obtain 80% power.

To compare cybersickness between the HMD-VR and control group, we tested the dependency of the occurrence of mild, moderate, severe and no physical complaints on the between-subject condition (HMD-VR versus control group) across all SSQ items using Bayesian contingency table tests^67^. The 95% credible interval of the difference in the probability of reporting a complaint between the HMD-VR and the control group was estimated using Markov Chain Monte Carlo sampling. Additionally, the proportion of participants who reported severe, moderate, mild or no discomfort for each SSQ item separately was calculated. In addition, we calculated the Frequentist Chi-square and Bayesian contingency table test for each item of the SSQ separately (Supplementary materials 2).

The number of verbal and manual interventions during the HMD-VR exposure were counted by one observer who was blind to the participant characteristics and test scores. These counts were based on audiotapes of the HMD-VR sessions.

All reported statistics are based on two-sided hypothesis tests and all BFs are based on uninformative prior distributions^63^. BFs larger than 3, which correspond to a 75% confidence level in the decision^68^, are interpreted as substantial evidence in favor of either the null or alternative hypothesis^44^ and BFs in between 2 (67% confidence level) and 3 are interpreted as anecdotal evidence^69^.

## Supplementary information

Supplementary materials
