# Supplementary material for: Acceptance of immersive head-mounted virtual reality in older adults
Source: Sci Rep. 2019 Mar 14;9:4519. doi: 10.1038/s41598-019-41200-6 (PMC6418153; doi:10.1038/s41598-019-41200-6)
Supplement: Supplementary file 1 — Supplementary materials [file 41598_2019_41200_MOESM1_ESM.pdf]

Supplementary materials 1

Acceptance of immersive head-mounted virtual reality in older adults

Hanne Huygelier , Brenda Schraepen, Raymond van Ee, Vero Vanden Abeele, and Céline R. Gillebert

Assumption check ANCOVA model

To assess whether the assumptions were violated for the analysis of covariance (ANCOVA), we visually inspected the association between the fitted values of the model and the residuals. The results are visualized in Figure S1a and Figure S1b. Figure S1a shows that the residuals are independent of the fitted values and shows no signs of heteroscedasticity or non-linearity. The histogram of the residuals of the ANCOVA shows no severe departures from normality (Figure S1b). These results show that there are no severe departures from the assumptions of homoscedasticity, linearity and normality.

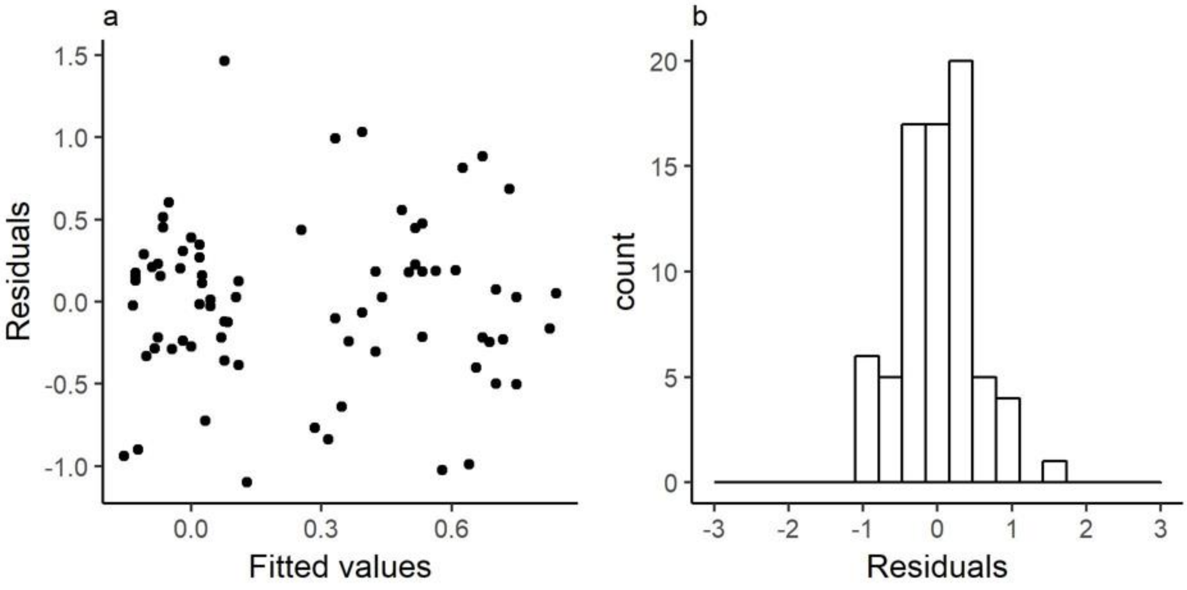

Supplementary Figure S1. (**a**) The residuals and fitted values. (**b**) The histogram of the residuals.

Supplementary materials 2

Acceptance of immersive head-mounted virtual reality in older adults

Hanne Huygelier , Brenda Schraepen, Raymond van Ee, Vero Vanden Abeele, and Céline R. Gillebert

Exploratory analyses on cybersickness symptoms

We tested whether there were significant group differences (HMD-VR versus control) on the single-symptom level of the cybersickness questionnaire. According to a factor analysis of the scale performed by Kennedy, Lane, Berbaum and Lilienthal (1993), the items of the scale can be subdivided in 3 subscales: nausea, oculomotor discomfort and disorientation[1]. Thus, the analysis was corrected for a total of 3 independent comparisons. Therefore, statistical significance was evaluated against an alpha level of 0.017, which approximates a Bayes Factor of 10.

We tested whether the complaints of cybersickness depended on the group (HMD-VR versus control) using a Frequentist Chi-square test and estimated the accompanying Bayes Factor by means of a Bayesian contingency table test[2]. The results of these analyses are reported in Supplementary Table S1. None of the symptoms show a statistically significant dependency on the group, not when p-values are compared to an alpha level of 0.017 (corrected for multiple comparisons), nor when p-values are compared to an alpha level of 0.05 (not corrected for multiple comparisons). Furthermore, the Bayes Factors all favor the null hypothesis.

Supplementary Table S1. Differences in cybersickness complaints by item.

Subscale	*Item description*	χ^2	*p*	BF_{10}	BF_{01}
Nausea	General discomfort	2.53	0.28	0.10	10.00
Oculomotor discomfort	Fatigue	1.30	0.52	0.10	10.00
Oculomotor discomfort	Headache	0.19	0.67	0.16	6.25
Oculomotor discomfort	Eyestrain	2.25	0.52	0.03	33.33
Oculomotor discomfort	Difficulty focusing	5.60	0.13	0.09	11.11
Nausea	Increase of salivation	3.04	0.22	0.04	25.00
Nausea	Sweating	4.94	0.18	0.06	16.17
Nausea	Nausea	0.00	0.99	0.10	10.00
Nausea	Difficulty concentrating	3.40	0.33	0.04	25.00
Disorientation	Fullness of the head	4.22	0.24	0.06	16.17
Oculomotor discomfort	Blurred vision	1.00	0.61	0.06	16.17
Disorientation	Dizziness with eyes open	0.00	0.99	0.10	10.00
Disorientation	Dizziness with eyes closed	3.04	0.22	0.04	25.00
Disorientation	Vertigo	3.04	0.22	0.04	25.00
Nausea	Stomach awareness	2.00	0.16	0.20	5.00
Nausea	Burping	0.00	0.99	0.10	10.00

Note. *BF_{10} represents the strength of evidence in favor of the hypothesis that there is a dependency of cybersickness complaints and group (HMD-VR versus control). BF_{01} represents the strength of evidence in favor of the null hypothesis.*

References

1. Kennedy, R. S., Lane, N. E., Berbaum, K. S. & Lilienthal, M. G. Simulator Sickness Questionnaire: An Enhanced Method for Quantifying Simulator Sickness. *Int. J. Aviat. Psychol.* **3**, 203–220 (1993).
2. Gunel, E. & Dickey, J. Bayes Factors for Independence in Contingency Tables. *Biometrika* **61**, 545–557 (1974).

Supplementary materials 3

Acceptance of immersive head-mounted virtual reality in older adults

Hanne Huygelier , Brenda Schraepen, Raymond van Ee, Vero Vanden Abeele, and Céline R. Gillebert

Validity of questionnaire responses

Introduction

Responses to questionnaires are known to be sensitive to various biases. First, questionnaires are typically not administered face-to-face to account for a potential social desirability bias. However, in our study we administered all questionnaires through means of a semi-structured interview in which special care was taken to explain each question to the participant until he or she understood the question. Through this method, we aimed to reduce the cognitive load to answer the questions and maximize the likelihood that participants understood the questions[1]. The latter was important to avoid a sampling bias in which mostly highly educated or cognitive healthy participants would respond to the questionnaires. However, this procedure may also introduce a social desirability bias. Therefore, it can be questioned whether the initial attitudes of older adults towards HMD-VR were truly neutral. For this reason, the Marlowe-Crowne social desirability scale[2] was administered to the last 44 individuals who participated in the study. If a social desirability bias affected the initial attitudes towards HMD-VR, a positive association was expected between the initial attitudes and the scores on the Marlowe-Crowne social desirability scale.

Second, even though the examiner took special care to ensure that all participants understood the questionnaire items, it may still have been the case that respondents with global cognitive impairment misunderstood the questionnaire items. Therefore, it is important to assess to what extent participants with global cognitive impairment have deviant response patterns as compared to cognitive healthy participants. According to a review of Van Vaerenbergh and Thomas[1], it can be expected that respondents who have difficulties understanding survey language adopt a tendency to use the middle response category of a rating scale, regardless of the item content (*mid-point response style*). The mid-point response style should result in a low between-item variance on a questionnaire. In addition, although only few studies have investigated the relation between cognitive load and response styles[1], it has been suggested that high cognitive load results in a response style in which the respondent agrees more often with statements than disagrees with statements (*net acquiescence response style*) or a response style in which respondents agree with statements regardless of the item content (*acquiescence response style*).

Net acquiescence response style leads to high between-item variance as respondents will either choose to disagree with statements or agree with statements, and will choose the middle response category less. Acquiescence response style results in low between-item variance. Thus, an extreme low or an extreme high between-item variance can be used as a marker of misunderstanding questionnaire items. To test whether participants with mild cognitive impairment had trouble understanding questionnaire items, we assessed whether patterns of extreme low or high between-item variance occurred among individuals with mild cognitive impairment and we evaluated whether there was an association of deviant response styles and global cognitive impairment.

Finally, it could be questioned whether measuring attitude change is reliable for participants with mild cognitive impairment. That is, participants with mild cognitive impairment may be less able to use their responses of the first administration of the attitude scale to calibrate their responses on the second administration of the attitude scale as compared to cognitive healthy respondents. Consequently, a low score on the Montreal Cognitive Assessment (MoCA) could result in less consistent responding across the two administrations of the attitude scale, resulting in attitude differences of a larger magnitude. For this reason, we evaluated the association between the absolute attitude differences and the MoCA scores. We used the absolute attitude difference, as we did not expect that MoCA scores would affect the direction, but only the magnitude of the attitude differences.

Method

1. ***Social desirability***

To evaluate whether a social desirability bias affected the initial attitudes towards HMD-VR, the Marlowe-Crowne social desirability scale[2] (SDS scale) was administered to the last 44 participants who were recruited (6 from the HMD-VR group and 38 from the control group – please note that the SDS scale was administered prior to the introduction of any group differences). The SDS scale contains 10 statements with which the participant can either agree or disagree. The more statements with which the participant agrees, the more socially desirable they responded. As we only have SDS data available in a subset of participants, we tested whether these two subgroups were representative of each other with regard to their initial attitudes. A Welch two sample t-test was used to test whether the group of participants with SDS data ($n = 44$) and the group of participants without SDS data ($n = 32$) significantly differed in initial attitudes. Accompanying Bayes Factors (BF) were computed with the Bayes Factors package[3]. Then, we estimated the Pearson correlation between the SDS scores and initial attitudes and calculated the accompanying BFs based on the method described in Wetzel and Wagenmakers[4].

2. *Response styles of participants with mild cognitive impairment*

We evaluated whether there was a systematic trend in the data suggesting that participants with mild cognitive impairment had difficulties answering the questions. A consistent pattern of extreme (high or low) between-item variance across different questionnaires using Likert scales was considered as an indicator of misunderstanding questionnaire items.

Quantifying the multivariate response styles

1. Questionnaire selection

First, we selected the questionnaires that were rated on a 5-point Likert scale. This selection was done, because we can expect more consistency in response styles across questionnaires with similar scales[1], and a higher consistency in response styles allows for a more sensitive detection of deviant response styles. Hence, the social desirability and the cybersickness questionnaire were left out of these analyses. In addition, we did not consider responses on the user experience scale or the second administration of the attitude scale as the HMD-VR and control group evaluated a different experience (HMD-VR exposure versus time-lapse videos) on these two scales. Consequently, the sample of participants evaluating the same experience on these two scales was too limited to detect deviant response styles. Thus, we evaluated the multivariate pattern of response styles on three questionnaires: the computer proficiency scale, the computer self-efficacy scale and the first administration of the attitude scale.

2. Calculating the multivariate response style

For each participant, the between-item variance on each of the three selected questionnaires (computer proficiency, computer self-efficacy and the initial attitude scale) was computed. Thus, for each participant we obtained three between-item variances, one for each scale. These three between-item variances constituted the multivariate response pattern of a participant.

Detecting deviant multivariate response styles

1. Estimating the expected multivariate response style

To diagnose extreme cases of multivariate response styles, we first established a normative multivariate response style. We assumed that individuals without cognitive impairment on the MoCA did not have systematic difficulties in understanding the questionnaire items. These individuals were used as the *normative group* to establish a normative multivariate between-item variance profile. Thus, all participants with a score above or equal to the cut-off score of 26 were used to establish the normative response profile (n = 33). Based on these data we calculated the average between-item variance on each questionnaire and the variance-covariance matrix of the between-item variances across questionnaires.

2. Quantifying the deviance of the multivariate response profiles

The extent to which the multivariate response profile of all participants with a MoCA score below 26 (*mild cognitive impairment*, MCI group) deviated significantly from the normative group, was calculated. For this purpose, a multivariate normative comparison was made based on the method and R code described by Huizenga et al.[5,6]. The multivariate normative comparison (MNC) statistic is calculated by comparing scores of an individual on multiple questionnaires to the mean scores of a normative group on these questionnaires and considers the covariance of these questionnaires in the norm group. The MNC statistic follows an F distribution with degrees of freedom p and n-p [5], where n represents the number of participants of the norm group and p represents the number of questionnaires.

Thus, for each participant with a score lower than 26 on the MoCA (n = 43) we computed the MNC statistic and calculated the probability that a value equal or more extreme would occur in the F distribution with 3 and 30 degrees of freedom. As this involved 43 independent statistical comparisons, we applied a Bonferroni correction on the alpha level, resulting in an alpha level of 0.0012.

3. Global cognitive status and attitude change

To test whether participants with mild cognitive impairment were less consistent in their responses to the two administrations of the attitude scale, we calculated the absolute value of the difference between the attitude score on the first and second administration of the attitude scale for each participant. Then, we estimated the Pearson correlation between the absolute attitude difference and the MoCA score of each participant. Additionally, we estimated the accompanying Bayes Factor of the correlation using the method described by Wetzel and Wagenmakers[4].

Results

1. Social desirability

The participants agreed on average with 60% of the 10 social desirability items (SD = 18%). The total sample (n = 76) had a mean initial attitude of 3.17 (SD = 0.76), the subgroup of which we have no SDS scores (n = 32) had a mean initial attitude of 3.35 (SD = 0.66) and the subgroup of which we have SDS scores (n = 44) had a mean initial attitude of 3.05 (SD = 0.80) (Figure S2a). There was no significant difference in attitudes between the two groups according to the Welch two sample t-test, t(72.84) = 1.79, P = 0.08, BF_{01} = 1.56. There was evidence in favor of a null correlation between social desirability and initial attitudes (r(42) = 0.08, P = 0.59, BF_{01} = 7.4, 95% CI [-0.22, 0.37]) (Figure S2b).

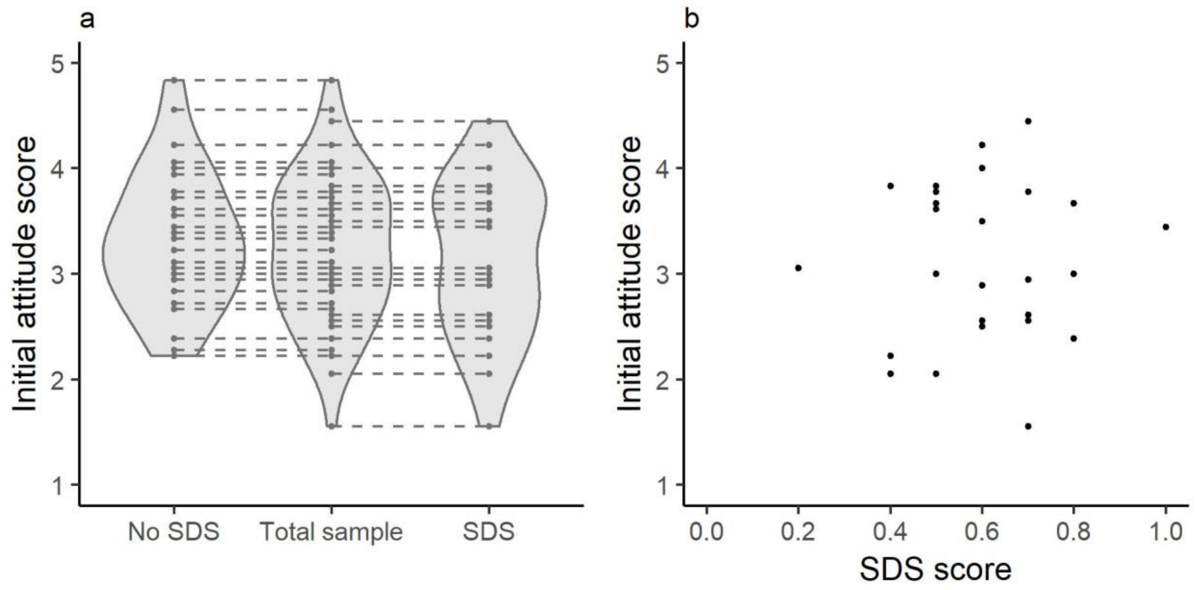

Supplementary Figure S2. (**a**) The initial attitudes of the participants in the group without SDS data (No SDS), the total sample and the subgroup with SDS data (SDS). The lines connect observations from the same participants. (**b**) The relation between the proportion of statements that the participant agreed with on the SDS scale (SDS score) and the initial attitude score.

2. Response styles of participants with mild cognitive impairment

The 3D scatterplots of the between-item variances on the three scales of the normative group and the MCI group are shown in Figure S3a. Figure S3a illustrates that the multivariate distributions of both groups are intermixed rather than clustered.

The results from the multivariate normative comparisons are presented in Figure S3b. Only one participant's multivariate response profile was significantly different from the normative group evaluated at an alpha level of 0.0012. That is, the MNC statistic was larger than 6.88 (indicated by the dashed line in Figure S3b) for one participant with a MoCA score of 22. In addition, we tested whether the MNC statistic, which quantifies the extent to which an individual deviated from the normative group, was associated to the MoCA scores in the MCI group. There was strong support for an absence of a correlation between the MNC statistic and the MoCA score, $r(41) = -0.05$, $p = 0.76$, 95% CI [-0.34, 0.26], $BF_{01} = 8.01$ (Figure S3b).

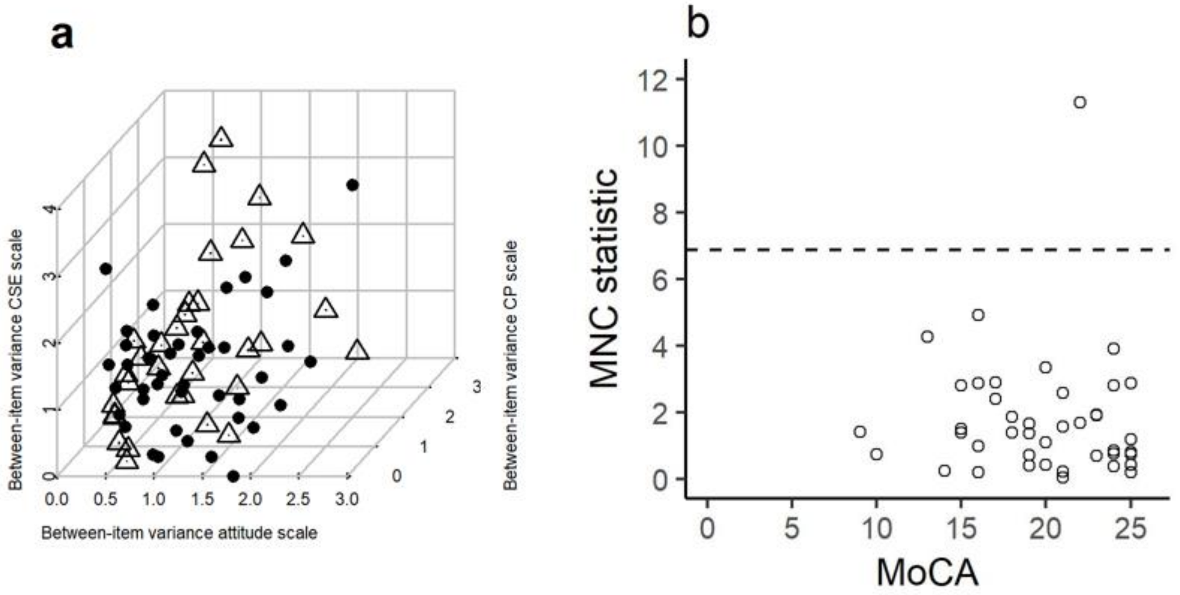

Supplementary Figure S3. (**a**) The multivariate distributions of the between-item variances on the three scales for the normative group who performed >= 26 on the MoCA (triangles) and for the MCI group who performed < 26 on the MoCA (dots). (**b**) The relation between the multivariate normative comparison statistics value (MNC statistic) and the MoCA score in the MCI group. The dashed line represents the cut-off value of the test statistic to evaluate a value as statistically significant corrected for multiple comparisons (alpha = 0.0012). CP = computer proficiency, CSE = computer self-efficacy.

3. Global cognitive status and attitude change

Across all participants, there was strong support for an absence of an association between the absolute attitude difference and the MoCA score, r(73) = 0.11, P = 0.33, 95% CI [-0.12, 0.33], BF_{01} = 6.80 (Figure S4).

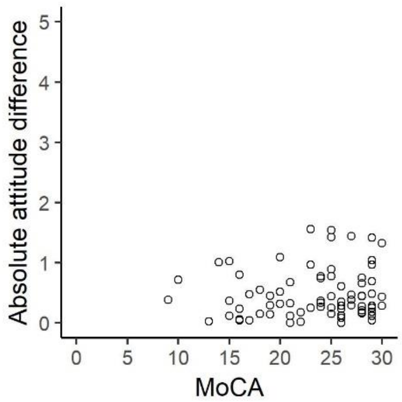

Supplementary Figure S4. The scatterplot of the absolute difference in attitudes on the first and second administration of the attitude scale (y-axis) and the MoCA scores (x-axis) of the 75 participants who completed the two administrations of the attitude scale.

Discussion

Results of survey studies are sensitive to different types of biases. Therefore, it is important to assess to what extent such biases may have affected the results of our study. For this reason, we checked whether a social desirability bias affected the initial attitudes towards HMD-VR, whether participants with mild cognitive impairment had deviant response styles (suggesting issues with understanding questionnaire items) and whether participants with mild cognitive impairment were equally consistent as cognitive healthy individuals in their responses on two administrations of the attitude scale.

The absence of an association between the SDS scale and the initial attitudes supports the idea that the initial attitudes towards HMD-VR were not biased by social desirability. Therefore, it is unlikely that a social desirability bias would explain why older adults initially had neutral attitudes towards HMD-VR. As there was no difference between the average initial attitudes between the subgroup that completed the SDS scale and the subgroup that did not complete the SDS scale, we conclude that the subgroup with SDS data was representative of the subgroup without SDS data regarding initial attitudes.

In addition, we found no difference in response styles between participants with low MoCA scores and participants with high MoCA scores. These results suggest that participants with low MoCA scores were equally able to understand the questionnaire items as the participants with high MoCA scores.

Moreover, there was no relation between the magnitude of the differences in self-reported attitudes towards HMD-VR between the two administrations of the attitude scale and the MoCA scores of participants. Thus, it seems that mild cognitive impairment did not compromise the test-retest results.

To conclude, we found no evidence that social desirability was related to initial attitudes towards HMD-VR. These results support the idea that older adults truly had neutral attitudes towards HMD-VR before they were exposed to HMD-VR. We also found no systematic differences in response styles between participants with mild cognitive impairment versus cognitive healthy participants. Thus, participants with mild cognitive impairment were equally able to understand the questionnaire items as participants without cognitive impairment. These results strengthen the validity of our findings and suggest that mild cognitive impairment did not hinder the validity of our survey research.

References

1. Van Vaerenbergh, Y. & Thomas, T. D. Response Styles in Survey Research: A Literature Review of Antecedents, Consequences, and Remedies. *Int. J. Public Opin. Res.* **25**, 195–217 (2013).
2. Strahan Robert & Gerbasi Kathleen Carrese. Short, homogeneous versions of the Marlow-Crowne Social Desirability Scale. *J. Clin. Psychol.* **28**, 191–193 (1972).
3. Rouder, J. N., Morey, R. D., Speckman, P. L. & Province, J. M. Default Bayes factors for ANOVA designs. *J. Math. Psychol.* **56**, 356–374 (2012).
4. Wetzels, R. & Wagenmakers, E.-J. A default Bayesian hypothesis test for correlations and partial correlations. *Psychon. Bull. Rev.* **19**, 1057–1064 (2012).
5. Huizenga, H. M., Smeding, H., Grasman, R. P. P. P. & Schmand, B. Multivariate normative comparisons. *Neuropsychologia* **45**, 2534–2542 (2007).
6. Agelink van Rentergem, J. A., de Vent, N. R., Schmand, B. A., Murre, J. M. J. & Huizenga, H. M. Multivariate normative comparisons for neuropsychological assessment by a multilevel factor structure or multiple imputation approach. *Psychol. Assess.* **30**, 436–449 (2018).

Supplementary materials 4

Acceptance of immersive head-mounted virtual reality in older adults

Hanne Huygelier, Brenda Schraepen, Raymond van Ee, Vero Vanden Abeele, and Céline R. Gillebert

Item selection and scale reliability

One item had an item-total correlation lower than 0.20 (Table S2) and was dropped from the attitude scale. One item was dropped from the experience scale according to this criterion (Table S3) and one item was dropped from the Neuroticism Extraversion Openness Five Factor Inventory 3 openness scale. None of the items had an item-total correlation lower than or equal to 0.20 on the computer proficiency or computer self-efficacy scale (Table S4, Table S5).

The attitude scale (after exclusion of item 8, Table S2) had an excellent internal consistency on the first administration (α = 0.91, 95% CI [0.88, 0.94]) and an average inter-item correlation of 0.36. The internal consistency on the second administration was higher (α = 0.95, 95% CI [0.93, 0.96]) and the average inter-item correlation was 0.47. The user experience scale (after exclusion of item 12, Table S3) had an excellent internal consistency (α = 0.87, 95% CI [0.82, 0.91]) and an average inter-item correlation of 0.25. The computer proficiency scale had an excellent internal consistency (α = 0.98, 95% CI [0.98, 0.99]) and an average inter-item correlation of 0.73. The computer self-efficacy scale had an excellent internal consistency (α = 0.94, 95% CI [0.92, 0.96]) and an average inter-item correlation of 0.53. The Neuroticism Extraversion Openness Five Factor Inventory 3 openness scale (after exclusion of 1 item) had a moderate internal consistency (α = 0.75, 95% CI [0.67, 0.83]) and an average inter-item correlation of 0.22.

Supplementary Table S2. Item statistics and descriptions of attitude scale.

Item ID	*Item description*	*R*	*M*	*SD*
1	Using virtual reality goggles would make me nervous. [1]	0.76	3.11	1.32
2	I am excited to use virtual reality. [1]	0.65	2.88	1.11
3	Using virtual reality would make me uncomfortable. [2]	0.67	3.08	1.25
4	I am looking forward to using virtual reality. [1]	0.70	2.96	1.46
5	I think that there are many usages for virtual reality goggles. [2]	0.52	3.88	0.83
6	I think that virtual reality is difficult to use. [1]	0.50	3.12	1.14
7	I think that virtual reality will have a big impact on the future. [2]	0.31	3.66	1.00
8	I think that few people are open to using virtual reality.	0.17	2.96	1.10
9	I think that I would like to use virtual reality often.	0.66	2.24	1.07
10	I think that I would have to learn a lot to use virtual reality.	0.37	2.42	1.30
11	I think that virtual reality is safe to use. [1]	0.32	3.46	0.96
12	I feel worried about using virtual reality. [2]	0.56	3.68	1.18
13	If I would have access to a virtual reality device, then I would want to use it.	0.67	2.93	1.27
14	I am reluctant to use virtual reality.	0.76	3.43	1.46
15	I believe that virtual reality can be used for different age groups.	0.55	3.57	1.15
16	I think that virtual reality is useless. [1]	0.57	3.64	1.24
17	I would recommend the use of virtual reality to friends and acquaintances.	0.53	2.76	1.08
18	I find virtual reality intimidating. [2]	0.65	3.12	1.21

Note. R = item-total correlation excluding the item itself. M = mean of scale without item. SD = SD of scale without item. [1] Items were based on Shaft, T. M., Sharfman, M. P. & Wu, W. W. Reliability assessment of the attitude towards computers instrument (ATCI). Comput. Hum. Behav. 20, 661–689 (2004). [2] Items were based on Durndell, A. & Haag, Z. Computer self efficacy, computer anxiety, attitudes towards the Internet and reported experience with the Internet, by gender, in an East European sample. Comput. Hum. Behav. 18, 521–535 (2002).

Supplementary Table S3. Item statistics and descriptions of user experience scale.

Item ID	*Item description*	*R*	*M*	*SD*
1	I have enjoyed the experience. [3]	0.71	4.44	0.81
2	I would describe the experience as interesting. [3]	0.66	4.43	0.74
3	I thought the experience was confusing.	0.43	4.28	1.03
4	I felt tense during the experience. [3]	0.33	4.00	1.30
5	I was interested in the experience. [4]	0.62	4.49	0.79
6	I thought the experience was boring. [3]	0.47	4.28	1.01
7	I thought that I could use the controllers easily.	0.21	3.25	1.36
8	I thought that the experience was frustrating.	0.53	4.44	0.89
9	It was important to me to do well during the experience. [3]	0.49	4.41	0.81
10	I would like to relive this experience.	0.78	3.93	1.17
11	I thought it was too difficult.	0.30	4.39	0.84
12	I felt as if I had little control over the experience. [4]	0.09	3.07	1.27
13	After the experience I felt as if I returned from a journey. [4]	0.55	3.11	1.38
14	I was disappointed that the experience was over. [4]	0.72	3.36	1.27
15	During the experience I lost track of time. [4]	0.25	3.20	1.43
16	The environment and objects in the environment did not seem real. [4]	0.26	3.95	1.11
17	I felt as if I was visiting the environment. [4]	0.53	3.69	1.19
18	I had more attention for the experience than for my own thoughts. [4]	0.31	4.29	0.83
19	I wanted the experience to be over as soon as possible. [4]	0.67	4.29	1.04
20	I thought that the environment did not look nice.	0.30	4.68	0.52
21	I felt as if the objects in the environment could touch me. [4]	0.27	2.79	1.47
22	I was curious to discover the environment.	0.57	4.35	0.88
23	I did not feel as if I was in the same place as the objects in the environment. [4]	0.32	3.36	1.46

Note. R = item-total correlation excluding the item itself. M = mean of scale without item. SD = SD of scale without item. [3] Items were based on McAuley, E., Duncan, T. & Tammen, V. V. Psychometric Properties of the Intrinsic Motivation Inventory in a Competitive Sport Setting: A Confirmatory Factor Analysis. Res. Q. Exerc. Sport 60, 48–58

(1989). [4] Items were based on Lessiter, J., Freeman, J., Keogh, E. & Davidoff, J. A Cross-Media Presence Questionnaire: The ITC-Sense of Presence Inventory. Presence Teleoperators Virtual Environ. 10, 282–297 (2001).

Supplementary Table S4. Item statistics and descriptions of computer proficiency scale.

Item ID	*Item description*	*R*	*M*	*SD*
5	I can turn a computer on and off. [5]	0.81	3.75	1.74
6	I can use a keyboard to type. [5]	0.77	3.50	1.65
7	I can use a computer mouse. [5]	0.82	3.68	1.80
8	I can change the volume on a computer.	0.88	3.21	1.86
9	I can change the size of text on a computer screen.	0.82	3.04	1.72
10	I can print a document.	0.93	3.11	1.82
11	I can add paper in the printer.	0.87	3.18	1.91
12	I can solve an issue when paper gets stuck in the printer.	0.79	2.66	1.69
13	I can open e-mails. [5]	0.94	3.24	1.91
14	I can send e-mails. [5]	0.94	3.17	1.91
15	I can send the same e-mail to multiple people at once. [5]	0.89	2.82	1.91
16	I can open documents that I received through e-mail. [5]	0.95	3.12	1.88
17	I can send pictures via e-mail.	0.85	2.58	1.84
18	I can use a search engine. [5]	0.92	3.14	1.90
19	I can find information about my hobbies online. [5]	0.94	3.05	1.86
20	I can read the newspaper online. [5]	0.89	3.09	1.93
21	I can buy things online. [5]	0.76	2.36	1.80
22	I can save a website in my favorites. [5]	0.82	2.46	1.73
23	I can save pictures from the internet on my computer.	0.85	2.49	1.75
24	I can use the computer to play games.	0.65	2.86	1.79
25	I can use the computer to watch a video.	0.67	2.08	1.59
26	I can use the computer to listen to music.	0.82	2.67	1.84

Note. R = item-total correlation excluding the item itself. M = mean of scale without item. SD = SD of scale without the item. [5] These items were retrieved from Boot, W. R. et al. Computer Proficiency Questionnaire: Assessing Low and High Computer Proficient Seniors. The Gerontologist 55, 404–411 (2015).

Supplementary Table S5. Item statistics and descriptions of computer self-efficacy scale.

Item ID	*Item description*	*R*	*M*	*SD*
1	I feel confident making selections from an on screen menu. [6]	0.77	3.05	1.39
2	I feel confident using the computer to write a letter or essay. [6]	0.80	3.11	1.63
3	I feel confident escaping or exiting a program or software. [6]	0.79	3.54	1.69
4	I feel confident calling up a data file to view on the monitor screen. [6]	0.85	3.25	1.64
5	I feel confident troubleshooting computer problems. [6]	0.74	2.04	1.12
6	I feel confident understanding terms or words relating to computer hardware. [6]	0.83	2.63	1.49
7	I feel confident explaining why a program (software) will or will not run on a given computer. [6]	0.77	2.21	1.19
8	I feel confident writing simple programs for the computer. [6]	0.49	1.45	0.90
9	I feel confident learning to use new technology.	0.61	2.84	1.23
10	Working with a computer makes me very nervous. [6]	0.66	3.46	1.44
11	Computers make me feel uncomfortable. [6]	0.62	3.64	1.33
12	Computers make me feel uneasy and confused. [6]	0.67	3.71	1.37
13	I think I can quickly learn how to use new technology. [6]	0.50	2.93	1.10
14	Which score would you give yourself on general computer skills?	0.82	2.20	1.15

Note. R = item-total correlation excluding the item itself. M = mean of scale without item. SD = SD of scale without item. [6] items were based on Barbeite, F. G. & Weiss, E. M. Computer self-efficacy and anxiety scales for an Internet sample: testing measurement equivalence of existing measures and development of new scales. Comput. Hum. Behav. 20, 1–15 (2004).
